# Evaluation of the potential of miR-21 as a diagnostic marker for oocyte maturity and embryo quality in women undergoing ICSI

**DOI:** 10.1038/s41598-023-28686-x

**Published:** 2023-01-25

**Authors:** Maryam Jenabi, Parvin Khodarahmi, Farzaneh Tafvizi, Saeed Zaker Bostanabad

**Affiliations:** Department of Biology, Parand Branch, Islamic Azad University, Parand, Iran

**Keywords:** Cell biology, Developmental biology, Genetics

## Abstract

MicroRNAs are small molecules that play a crucial role in regulating a woman's reproductive system. The present study evaluates the expression of miR-21 in the serum, follicular fluid (FF), and cumulus cells (CCs) and their association with oocyte maturity and embryo quality in women undergoing intracytoplasmic sperm injection. Women subjects were divided into the case (54 Patients with female factor infertility) and control groups (33 patients with male factor infertility). The level of miR-21 was measured using Real-Time PCR. The level of miR-21 was significantly lower in the CCs, FF, and serum in the case compared to the control group (*p* < 0.05). MiR-21 abundance was higher in FF and CCs samples than in serum. Furthermore, there was a significant increase in CCs to FF in the case group (*p* < 0.05). A significant decrease in oocyte count, MII oocytes, and percentage of mature oocytes were observed in the case group (*p* < 0.05). The expression of miR-21 in FF and CCs was positively related to oocyte maturation, but no correlation with embryo development was observed. This study found that miR-21 is expressed less in women with female factor infertility, and human oocytes' development is crucially affected by the expression of miR-21. Therefore, miR-21 could provide new helpful biomarkers of oocyte maturity.

## Introduction

MicroRNAs (miRNAs) are single-stranded RNA molecules with nearly 19–25 nucleotides^1^. These RNAs work by regulating and controlling gene expression after transcription or post-transcriptional levels at the mRNA level. miRNAs bind specifically to mRNA and inhibit gene expression through translational repression or degradation of mRNA^1,2^. Most miRNAs can regulate hundreds of genes at once. However, one target gene may be controlled by more than one miRNA^3^. In the human genome, miRNAs regulate 30% of all active genes^2^.

In reproductive health, miRNAs are involved in physiological and pathological processes in mammals and humans^4,5^. Various reproductive biological processes and signaling pathways are influenced by MiRNAs, including cell proliferation, apoptosis, steroidogenesis, oocyte growth, oocyte maturation, embryo development, and pregnancy^6–9^. It was demonstrated that miRNAs play a role in oocyte and embryo development and could be used to identify diagnostic biomarkers and therapeutic targets^10–13^. They were recently revealed to contribute significantly to oogenesis and embryogenesis^14,15^. It has been found that approximately 2000 miRNA genes are differentially expressed in ovarian follicles during oogenesis^16^. The process of oogenesis is complex; it involves interaction between a growing oocyte and the surrounding cells within its follicular microenvironment. Ovarian granulosa cells (GCs) are vital somatic cells that support follicular growth and oocyte development by sharing paracrine factors^17^. MiRNAs are expressed in GCs and CCs, and their expression may have influenced GC fate, such as proliferation, differentiation, and apoptosis^18,19^. Moreover, the reproductive tissues and biological fluids, such as FF, are composed primarily of miRNAs^20–22^. The FF is formed by the secretion and metabolic activity of oocytes, GCs, CCs, and theca cells, as well as the transport of blood plasma components. Oocyte growth and maturation depend highly on this fluid because it provides a suitable intra-follicular environment^16,23^. The studies indicated that oocyte competence and embryo development influenced the FF. Therefore, it is believed that differences in its composition can predict the quality of oocytes and embryos^24,25^. The FF contains miRNAs that are produced by oocytes and their surrounding cells. It is possible that changes in the miRNA profile in the FF influence oocyte maturation^8^. Furthermore, some circulating miRNAs in FF could be used to develop a highly personalized IVF strategy through non-invasive, powerful means of predicting blastulation and clinical pregnancy outcomes^26^. Therefore, understanding the regulatory mechanisms mediated by miRNAs during oogenesis and embryogenesis can help understand the molecular mechanisms that lead to oocyte formation, early embryo development, and implantation^27^.

MiR-21 belongs to the miRNA family, which can regulate a wide range of genes^28^. It is one of the most abundant miRNA in bovine and human CCs^8,29^. Studies have demonstrated that miR-21 is expressed in murine, bovine, pig, and human CCs during oocyte maturation and embryonic development^8,29–33^. It acts as an anti-apoptotic factor in GCs and losing it reduces ovulation rates in vivo^33^. It is demonstrated that miR-21 expression is changed in polycystic ovary syndrome (PCOS) patients. Moreover, GCs of PCOS patients express different levels of miR-21, which affects follicle health and growth^34–36^.

This study aimed to determine whether miR-21 can be differentially expressed in serum, FF, and CCs in women with female factor infertility. Furthermore, can miR-21 be used to evaluate oocyte maturity and embryo quality?

## Results

### Evaluation of different biochemical parameters

A total of 33 patients with male factor infertility diagnoses with an average age of 29 years and a BMI of 21.9 participated in the study in the control group. Subjects in the case group were 54 patients with an average age of 31 years and a BMI of 22.6 with differing female factor infertility. There were no considerable differences in age or BMI between the case and control groups (*p* = 0.180 and *p* = 0.886, respectively).

The comparison of parameters, such as hormonal features and characteristics, is summarized (Table 1). The case and control groups did not significantly differ in FSH, LH, E2, Prolactin, TSH, and AMH (*p* > 0.05).

**Table1:** Comparison of serum hormonal evaluation in women undergoing ICSI in the case and control groups.

Parameters	Control group n = 33	Control group CV%	Case group n = 54	Case group CV%	*p* Value
FSH (IU/L)	6.23 ± 0.18	16.88	6.55 ± 0.33	38.00	0.401
LH (IU/L)	5.94 ± 0.17	16.67	6.10 ± 0.32	39.63	0.665
E2 (pg /ml))	42.83 ± 1.16	15.66	40.02 ± 1.86	34.20	0.205
TSH (m IU/L)	2.58 ± 0.07	17.27	2.63 ± 0.06	17.66	0.638
PRL (ng/ml)	13.29 ± 0.41	17.81	14.24 ± 0.57	29.45	0.183
AMH	2.85 ± 0.08	17.91	3.01 ± 0.27	67.84	0.588

Different parameters were compared between women in the case and the control groups who underwent ICSI. There were no significant differences in the case from the control group based on the T-test (*p* > 0.05). Follicle stimulating hormone (FSH), luteinizing hormone (LH), estradiol (E2), thyroid-stimulating Hormone (TSH), prolactin (PRL), anti mullerian hormone (AMH), coefficient of variation (CV%). The results are presented as the mean ± SEM.

### Evaluation of oocytes and embryos

**Figure 1:**
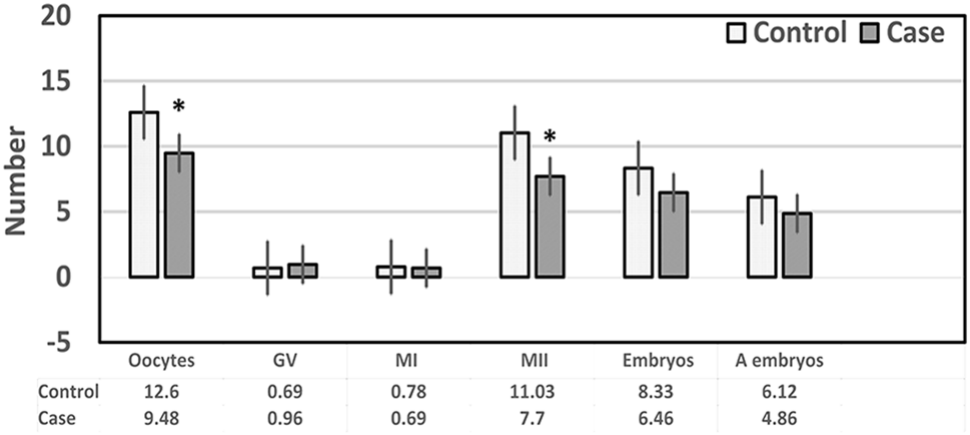
Comparison of different parameters between case and control groups. The number of obtained oocytes, GV, MI, and MII oocytes, the number of embryos, and A-grade embryos were evaluated between the case and control group based on the t-test, *p < 0.05. The results are presented as the mean ± SEM. *GV*: Germinal Vesicle oocyte, *MI*: Metaphase I oocyte, *MII*: Metaphase II oocytes, *A Embryo*: A-grade embryos.

A comparison of the number of oocytes produced, the GV, MI, and MII oocytes, the oocyte maturation rate, the count of embryos obtained, A-grade embryos, and the ratio of good-quality embryos was made between the case and the control groups. There were noticeably fewer obtained oocytes and MII oocytes in the case group (*p* < 0.05). In contrast, there was not a discernible difference between the case group and the control group in terms of the mean values of GV and MI oocytes, the number of embryos obtained, and A-grade embryos in the case group versus the control group (*p* > 0.05) (Fig. 1).

The oocyte maturation rate significantly decreased in the case group than in the control group (79.81 ± 3.10 vs. 86.98 ± 1.58, respectively, *p* = 0.043). However, there was no significant difference in the percentage of good-quality embryos in the case and control groups (78.49 ± 3.62 vs. 74.77 ± 4.80, respectively, *p* = 0.532).

These parameters were evaluated in four subgroups of the case group. A significant difference in oocytes obtained, MII oocytes, and the number of embryos obtained was observed between infertile women with ovarian dysfunction and uterine lesions factor (*p* < 0.05). Women with ovarian dysfunction factors have significantly lower oocyte production and maturation parameters because of ovarian inefficiency. The other parameters were not significant among the four subgroups (*p* > 0.05) (Table 2).

**Table 2:** Comparison of different parameters in four subgroups of the case group.

Parameters	Ovarian dysfunction n = 17	PCOS n = 16	Uterine lesions n = 8	Endometriosis n = 13	*p* Value
No. of oocytes obtained	6.06 ± 1.22	11.06 ± 2.13	16.38 ± 3.43	8.77 ± 1.95	a: 0.215
					b: 0.005*
					C: 0.809
					d: 0.366
					e: 0.891
					F: 0.097
No. of GV oocytes	0.47 ± 0.15	1.81 ± 0 58	1.71 ± 1.04	0.23 ± 0.16	a: 0.065
					b: 0.161
					C: 0.991
					d: 1.00
					e: 0.083
					F: 0.090
No. of MI oocytes	0.53 ± 0.15	1.00 ± 0.20	0.71 ± 0.71	0.62 ± 0.24	a: 0.648
					b: 0.624
					C: 0.999
					d: 0.998
					e: 0.834
					F: 0.779
No. of MII oocytes	5.06 ± 1.12	8.25 ± 1.78	13.25 ± 2.61	7.92 ± 1.86	a: 0.534
					b: 0.015*
					C: 0.683
					d: 0.299
					e: 1.00
					F: 0.273
Oocyte maturation%	82.72 ± 4.31	71.80 ± 6.15	82.37 ± 6.95	83.42 ± 7.57	a: 0.450
					b: 1.00
					C: 1.00
					d: 0.685
					e: 0.458
					F: 1.00
No. of embryos	4.41 ± 1.12	6.53 ± 1.14	11.88 ± 2.19	6.75 ± 1.71	a: 0.722
					b: 0.004*
					C: 0.695
					d: 0.091
					e: 1.00
					F: 0.142
Embryo quality%	89.85 ± 6.16	74.88 ± 4.52	65.81 ± 10.57	74.09 ± 8.93	a: 0.326
					b: 0.096
					C: 0.394
					d: 0.884
					e: 1.00
					F: 0.934

Different parameters were compared between four subgroups of the case group, including Ovarian dysfunction, PCOS, Uterine lesions, and Endometriosis. The sing * indicates a significant difference on the Anova followed by the Tukey HSD post Hoc test (*p < 0.05). a: *p* value between Ovarian dysfunction and PCOS groups, b: *p* value between Ovarian dysfunction and Uterine lesions groups, c: *p* value between Ovarian dysfunction and Endometriosis groups, d: *p* value between PCOS and Uterine lesions groups, e: *p* value between PCOS and Endometriosis groups, f: *p* value between Uterine lesions and Endometriosis groups. The results are presented as the mean ± SEM. *GV* germinal vesicle oocyte, *MI* metaphase I oocyte, *MII* metaphase II oocytes.

### Evaluation of miR-21 expression in the serum, CCs, and FF

**Figure 2:**
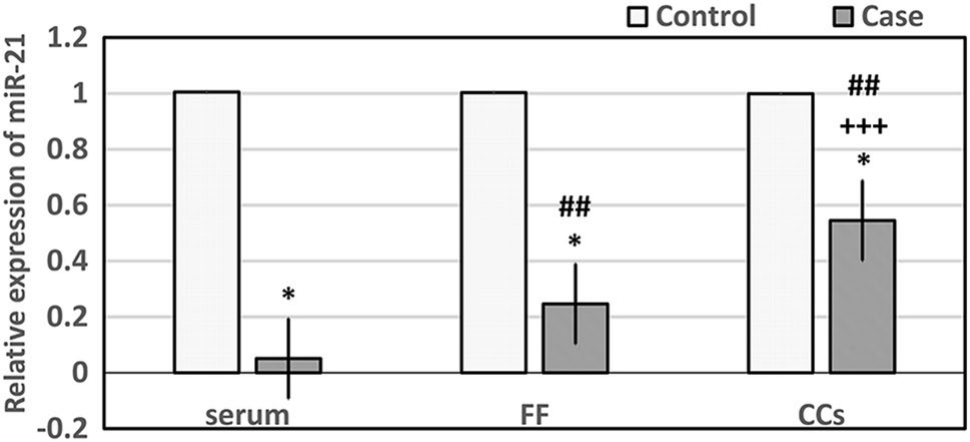
Comparison of miR-21 expression between the case and control groups. The level of miR-21 expression was significantly lower in the serum, FF, and CCs samples in the case group compared to the control group based on the t-test, *p < 0.05. The comparison of expression of miR-21 among three media (serum, FF, and CCs samples) in pairs indicated that the level of miR-21 expression was significantly higher in the FF and CCs than in the serum in the case group on the Anova test, ##*p* < 0.05. The miR-21 expression was significantly increased in the CCs than in the FF in the case group on the Anova test, +++*p* < 0.05. The results are presented as the mean ± SEM. *FF*: Follicular Fluid, *CCs*: Cumulus Cells.

The miR-21 expression was significantly lower in the FF, CCs, and serum samples in the case group than in the control group (*p* < 0.05). The serum, FF, and CCs samples were compared for miR-21 expression levels in pairs. The results exhibited a significant increase in the level of miR-21 expression in the FF and CCs samples than in the serum (*p* = 0.001 and *p* = 0.000, respectively). Additionally, there was a significantly higher level of miR-21 expression in the CCs than in the FF sample (*p* = 0.000) (Fig. 2).

The expression of miR-21 in the FF, CCs, and serum in the four subgroups of the case, including women with PCOS, endometriosis, uterine and cervical lesions, and ovarian dysfunction group, were compared with each other. According to the results, the relative expression of miR-21 was not significant between all groups (*p* > 0.05) (Table3).

**Table 3:** Comparison of miR-21 expression in four subgroups of the case group.

Parameters	Ovarian dysfunction (n = 17)	PCOS (n = 16)	Uterine lesion s (n = 8)	Endometriosis (n = 13)	*p* Value
Expression of miR-21 in the serum	0.053 ± 0.005	0.055 ± 0.005	0.045 ± 0.009	0.049 ± 0.007	a: 1.000
					b: 1.000
					C: 1.000
					d: 1.000
					e: 1.000
					F: 1.000
Expression of miR-21 in the FF	0.559 ± 0.271	0.117 ± 0.035	0.269 ± 0.091	0.326 ± 0.068	a: 0.238
					b: 0.801
					C: 0.838
					d: 0.978
					e: 0.889
					F: 1.000
Expression of miR-21 in the CCs	0.575 ± 0.112	0.328 ± 0.078	0.615 ± 0.195	0.685 ± 0.109	a: 0.466
					b: 1.000
					C: 0.957
					d: 0.535
					e: 0.179
					F: 0.996

Relative miR-21 expression was compared in the serum, FF, and CCs samples in the four subgroups of the case group (ovarian dysfunction, PCOS, uterine lesions, and endometriosis). Relative expression of miR-21 was not significant between all groups based on the Anova test (*p* < 0.05). a: *p* value between ovarian dysfunction and PCOS groups, b: *p* value between ovarian dysfunction and Uterine lesions groups, c: *p* value between ovarian dysfunction and Endometriosis groups, d: *p* value between PCOS and Uterine lesions groups, e: *p* value between PCOS and Endometriosis groups, f: *p* value between Uterine lesions and Endometriosis groups. The results are presented as the mean ± SEM. *FF* follicular fluid, *CCs* cumulus cells.

### Association between miR-21 expression with oocyte maturity and embryo quality

**Figure 3:**
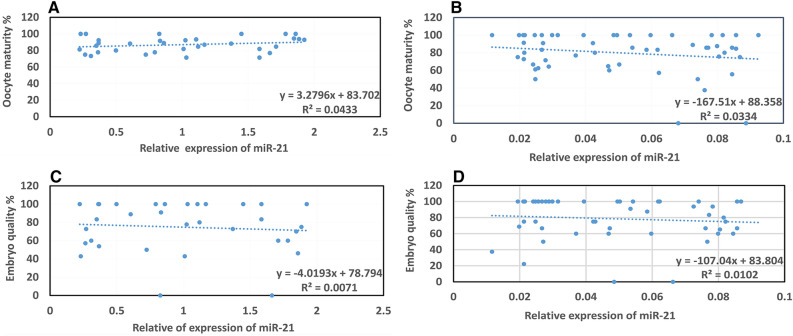
Correlation of the level of miR-21 expression in the serum sample with oocyte maturation (%) and embryo quality (%) in the case and control groups. There was no significant relationship between the rates of miR-21 expression in the serum with oocyte maturity and embryo quality in the case and control groups based on Pearson's correlation (*p* > 0.05). The results are presented as the mean ± SEM. (**A**) Correlation of the level of miR-21 expression in the serum sample with oocyte maturation in the control group. (**B**) Correlation of the level of miR-21 expression in the serum sample with oocyte maturation in the case group. (**C**) Correlation of the level of miR-21 expression in the serum sample with embryo quality in the control group. (**D**) Correlation of the level of miR-21 expression in the serum sample with embryo quality in the case group.

The link between the expression of miR-21 with oocyte maturity and embryo quality was studied using a Pearson's correlation test. Evaluation of the rates of miR-21 expression in the serum indicated no significant relationship to the oocyte maturity (*p* = 0.186 and *p* = 0.245, respectively) and embryo quality (*p* = 0.486 and *p* = 0.641, respectively) in the case and control groups (Fig. 3A–D).

**Figure 4:**
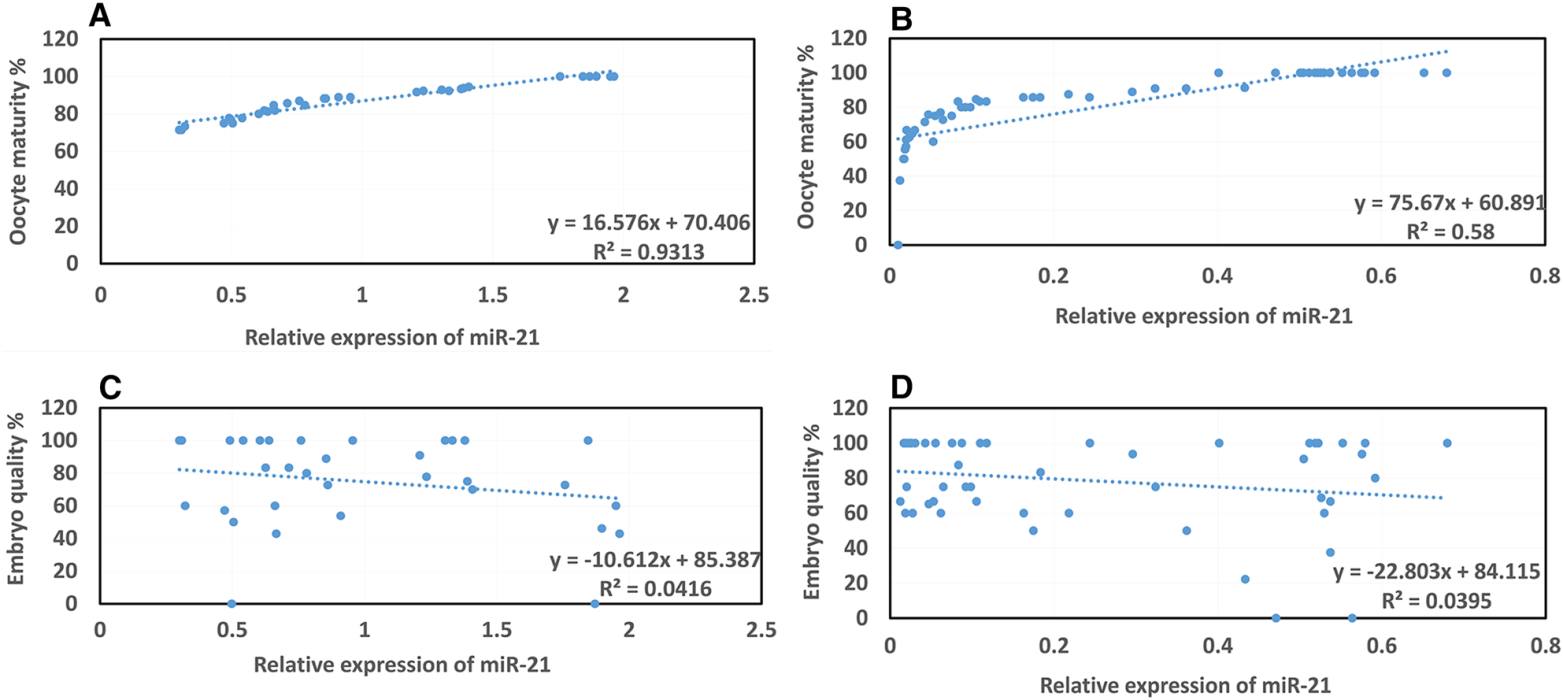
Correlation of the level of miR-21 expression in the follicular fluid (FF) with oocyte maturation (%) and embryo quality (%) in the case and control groups. The rate of miR-21 expression in the FF environments was directly related to the oocyte maturation in both groups based on Pearson's correlation (*p* < 0.05). There was no significant relationship between the rates of miR-21 expression in the FF and embryo quality based on Pearson's correlation (*p* > 0.05). The results are presented as the mean ± SEM. (**A**) Correlation of the level of miR-21 expression in the FF sample with oocyte maturation in the control group. (**B**) Correlation of the level of miR-21 expression in the FF sample with oocyte maturation in the case group. (**C**) Correlation of the level of miR-21 expression in the FF sample with embryo quality in the control group. (**D**) Correlation of the level of miR-21 expression in the FF sample with embryo quality in the case group.

The findings indicated that oocyte maturity was positively correlated with the frequencies of miR-21 expression in the FF in the case and control groups (*p* = 0.000 and *p* = 0.000, respectively). However, there was no significant relationship with embryo quality (*p* = 0.166 and *p* = 0.255, respectively) (Fig. 4A–D).

**Figure 5:**
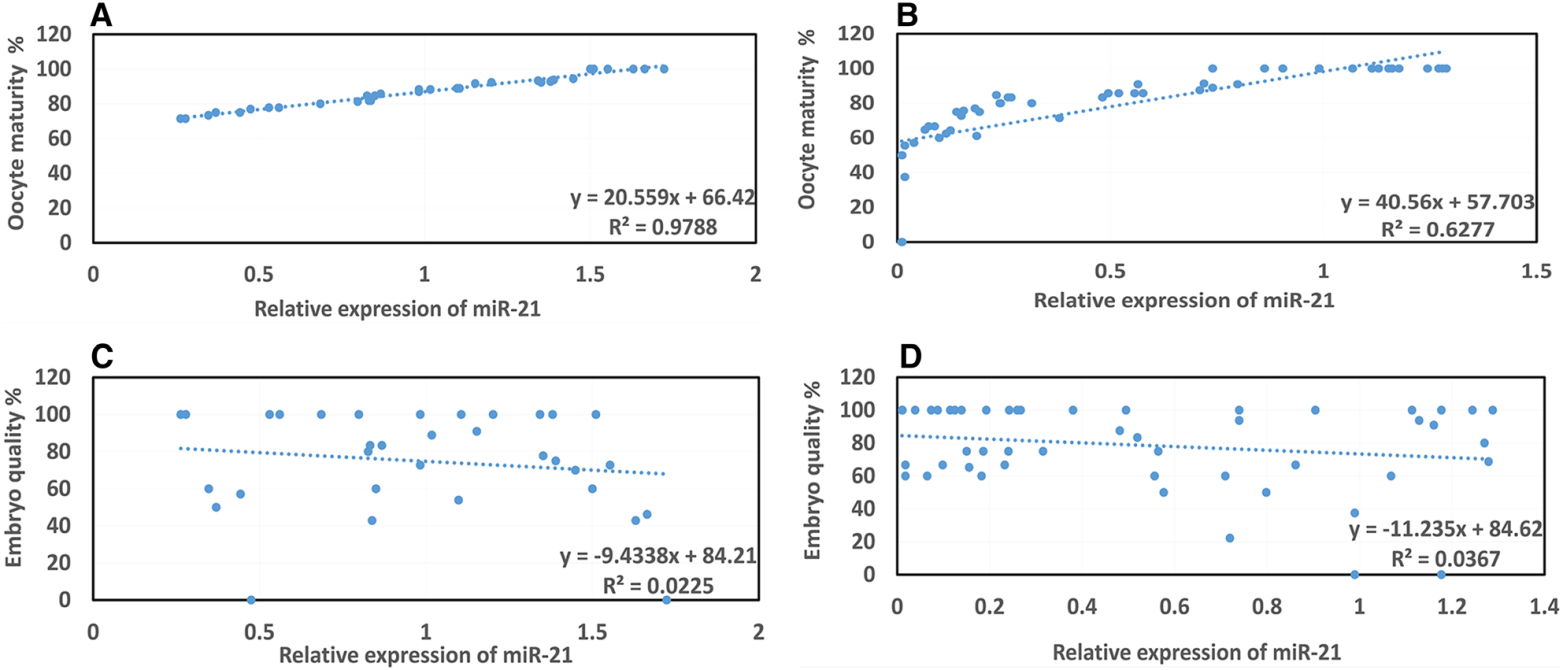
Correlation of the level of miR-21 expression in the cumulus cells (CCs) with oocyte maturation (%) and embryo quality (%) in the case and control groups. The rate of miR-21 expression in the CCs was directly related to the oocyte maturation based on Pearson's correlation (*p* < 0.05). There was no significant relationship between the rates of miR-21 expression in the CCs and embryo quality based on Pearson's correlation (*p* > 0.05). The results are presented as the mean ± SEM. (**A**) Correlation of the level of miR-21 expression in the CCs sample with oocyte maturation in the control group. (**B**) Correlation of the level of miR-21 expression in the CCS sample with oocyte maturation in the case group. (**C**) Correlation of the level of miR-21 expression in the CCs sample with embryo quality in the control group. (**D**) Correlation of the level of miR-21 expression in the CCs sample with embryo quality in the case group.

The evaluation level of miR-21 expression in the CCs expressed a significant association between CCs miR-21 and oocyte maturity in the case and control groups (*p* = 0.000 and *p* = 0.000, respectively). Despite this, neither group demonstrated a significant relationship with embryo quality (*p* = 0.183 and *p* = 0.405) (Fig. 5A–D).

### Discussion

There is evidence that miRNAs are crucial to reproductive diseases^37^. MiRNAs are expressed in CCs, FF, and embryos and regulate ovulation, and embryo development by modulating the expression of numerous genes^38^. There are 332 miRNAs with more than ten reads and 240 miRNAs with more than 50 reads on average in CCs. MiR-21 was one of CCs' most highly expressed miRNAs^29^. The present study was the first report that simultaneously investigated the correlation of the miR-21 expression in the CCs, FF, and serum with oocyte maturity and embryo quality in female factor infertility patients.

According to this study, hormone levels did not differ significantly between subfertile women and fertile women who underwent ICSI. Some factors, such as endometriosis, PCOS, ovarian dysfunction, and uterine and cervical lesions, caused infertility in the subfertile (case) group. In contrast, only male factors caused infertility in the fertile (control) group. There are variations in hormonal levels among subfertile women depending on the cause of their infertility. For example, as expected, low AMH levels (0.17–1.92 ng/ml) and high FSH levels (4.55–12 IU/L) were observed for women with ovarian dysfunction. In contrast, the PCOS patients had higher levels of AMH (2.93–6.91 ng/ml) and lower levels of FSH (3.11–6.69 IU/L) than other subgroups. Generally, PCOS patients had high levels of PRL and LH hormones. Therefore, the case group's hormone value reflects the average hormone level across the four subgroups, which was not significantly different from the control group.

The female factor infertility patients had significantly lower levels of miR-21 expression than fertile women undergoing ICSI. It appears that miR-21 reduction is related to women’s infertility. The studies on miRNAs in infertile women have been mainly focused on PCOS patients^19,27,28,35^. Yu et al.^28^ indicated that the miR-21 expression in granulosa cells was significantly increased in PCOS patients compared to the normal patient (tubal or male factor infertility). The proliferation rate of GCs significantly increased in the PCOS group after miR-21 overexpression, while the apoptosis rate decreased. It is proven that miR-21 can regulate cell proliferation, migration, cell cycle, and other biological functions^28^. Jiang et al.^34^ demonstrated a higher level of miR-21 expression in the serum of PCOS patients. According to them, miR-21 can act as a non-invasive biomarker for PCOS diagnosis^34^. On the contrary, Naji et al.^36^ revealed an increase in miR-21 levels in GCs and a decrease in miR-21 levels in their FF of PCOS patients. Among serum samples, miR-21 levels were not significantly different between the PCOS and control groups^36^. In our results, the level of the miR-21 expression was significantly reduced in serum, FF, and CCs in all subgroups of the case and PCOS group (The miR-21 levels were in the PCOS group: serum: 0.05 ± 0.005, FF: 0.11 ± 0.03 and CCs: 0.32 ± 0.07, respectively). There is conflicting information regarding the function of miR-21 in PCOS patients. Overall, with studies on its upregulation or downregulation properties, miR-21 may have different functions in different physiologic states and pathological conditions^35,39–41^.

The studies examined the expression of specific miRNAs during oocyte maturation and the early stages of embryo development. Based on the research findings, the miR-21 expression in bovines found an overall positive correlation evident from the GV stage up to the 8-cell embryo, with an increase from the MII stage to the 2-cell embryo^42^. In one study on pigs, Wright et al.^33^ found that miR-21 levels increased 25-fold during oocyte maturation, which regulated meiotic maturation. Gilchrist et al.^43^ indicated that miR-21 levels increased as CCs and oocytes progressed through the GV to MII stages of meiosis in domestic and laboratory animals. In agreement with these studies, our results demonstrated that the maturation of the oocyte increased with increasing the miR-21 concentrations in the FF and CCs. It could be concluded that miR-21 plays a crucial role in oocyte maturation. The correlation between miR-21 expression and oocyte maturation can be supported by the opinion that inhibition of miR-21 expression using miR-21 antisense oligonucleotides affects the expression of programmed cell death proteins and consequently suppresses oocyte maturation and embryo development at the cleavage stage^8,33^. The mechanisms explaining how miR-21, synthesized by CCs or other cells, affects oocyte development are different. There is a possibility that some of them may be responsible for the results of this study. There has been evidence that the survival of CCs is essential for oocyte viability, and miR-21 may have an impact on oocyte viability by suppressing genes that promote apoptosis in CCs^31,44–46^. It was found that treatment of CCs with an inhibitor of miR-21 increased the apoptosis mechanism mediated by a rise in PTEN expression, a gene known to inhibit the Akt-dependent survival pathway in CCs^47^. PTEN causes apoptosis by inhibiting the PI3/AKT kinase survival pathway. Therefore, increased miR-21 levels would probably reduce PTEN levels, increase AKT activity, and maintain the viability of CCs^48^.

The next part of our study focused on the evaluation of embryo development; there was no difference in the number of embryos obtained or A-grade embryos in the case group versus the control group. Furthermore, miR-21 expression was not correlated with embryo quality on the third day of development. Our findings are consistent with those obtained by Uhde et al.^29^, who indicated a similar expression of miRNA patterns in bovine CCs that did not cleave and CCs that became blastocysts. They concluded that the whole miRNA expression, including miR-21 in CCs, could not be used as a quality marker^29^. Inconsistent with our results, Dehghan et al.^49^ proposed that miR-21 increased zygotes' growth and embryo development in mice. The inhibition of miR-21 by miR-off 21 results in reduced blastocyst formation by altering the gene expression of Cdk2ap1 and Oct4. Furthermore, Bartolucci et al.^47^ found a significantly higher level of miR-21 in CCs associated with oocytes that became blastocysts compared to those that were arrested in the cleavage stage in 25 patients. Differences in the results can be attributed to the difference between humans and animals. Moreover, the other factors influence embryo development. Sperm quality is a factor influencing embryo development^50^.

According to our findings, the expression of miR-21 in the FF and CCs increased more than that in the serum in subfertile women. Interestingly, the miR-21 expression in FF correlated positively with it in CCs. MiRNAs are synthesized by CCs and other cells like mural granulosa cells. MiR-21 is transported from CCs to oocytes via extracellular vesicles until connections between CCs and oocytes are broken during ovulation^51^. The extracellular matrix and gap junctions enable communication between oocytes and CCs. The gap junctions facilitate the transfer of molecules from oocytes to CCs and regulate folliculogenesis^52^. It was demonstrated that the transfer of miR-21 occurs from CCs to oocytes during in vitro maturation. Since RNA transcription is suppressed during oocyte maturation, miR-21 levels do not correlate with the synthesis in the oocyte^53^. The presence of miRNAs in FF in membrane-bound microvesicles (exosomes) and soluble forms is well established^23,25^. A further characteristic of FF is that miRNA expression is highly stable, and it can accumulate during the development of the follicle^23^. The expression level of miRNAs in FF reflects the overall alternations in any diseased condition. Hence, FF's miRNA profile can exhibit more disparity than CCs^16,19,52^. According to the results in our study, miR-21 levels were lower in FF than in CCs. Several studies have been conducted on the mechanisms of action of miR-21 in CCs. Gilchrist et al.^54^ demonstrated that miR-21 expression was regulated in the CCs via the growth differentiation factor 9 (GDF9), which is secreted by the oocyte. It was documented that the correlation between miR-21 and GDF9 in cultured CCs was in the presence or absence of oocytes. MiR-21 levels were significantly lower in CCs without oocytes than in CCs with intact oocytes. The level of miR-21 in CCs without oocytes was returned to those in intact COCs when GDF9 was added to the media^31^. GDF9 binds with CCs receptors and activates the Small Mothers Against Decapentaplegic (SMAD)^55^. SMAD proteins likely promote miR-21 processing by increasing the Ribonuclease III (DICER) expression, resulting in the maturation of pre-miR-21^31,47^. In the ovaries, SMAD proteins regulate vital biological processes, thereby activating downstream molecules and modulating target gene expression. Our results suggest that changes in SMAD expression can cause reproductive process disorders such as PCOS and ovary dysfunction^56^. Other methods, like bone morphogenetic protein 15 (BMP15), affect the expression of miR-21 by GDF9. Abir and Fisch^57^ discovered that miR-21 overexpression could affect nuclear and cytoplasmic maturation by targeting BMPR2 as a receptor for BMP15. In addition, BMP-15 binds with BMPR1 and BMPR2 and mediates the SMAD signaling pathway. BMP-15 directly affects the functions of GCs, including steroidogenesis, inhibition of luteinization, differentiation of the cumulus, ovulation, and oocyte maturation^57^. A synergistic relationship between GDF9 and BMP15 leads to similar interactions. GDF9 suppresses apoptosis in CCs by increasing miR-21 levels via BMPR1, BMPR2 and transforming growth factor B receptors (TGFBR1). A low level of GDF9 and BMP-15 were found in the FF of follicles with a reduced oocyte developmental capacity^31^. More studies are needed to investigate these pathways in patients with female factor infertility.

In conclusion, the results indicated the significantly lower expression of miR-21 in serum, FF, and CCs of subfertile women undergoing ICSI. This reinforces the notion of reduced miR-21 involvement in the pathogenesis of infertile women with female factor infertility. The results demonstrated that the level of miR-21 expression in FF and CCs was directly associated with oocyte maturation. The relative presence of miR-21 in CCs and FF may help to predict oocyte maturation.

## Methods

### The selection of patients

Our Ethics Committee in Human Research approved the current study (IR.IAU.VARAMIN.REC.1399.035) and complied with the Declaration of Helsinki and the Health Insurance Portability and Accountability Act. All methods were performed in accordance with relevant guidelines and protocol of Parand branch, Islamic Azad University, Parand, Iran. Each couple undergoing the ICSI cycle signed a written consent form for both study participation and publication of information at the Academic Center for Education, Culture, and Research (ACECR), Infertility Center, Arak, Iran.

This prospective study was performed on 200 women undergoing ICSI in the age range of 20–35 years and with a standard body mass index (BMI < 25). Study participants were excluded if they took fertility supplements, had infertility due to mixed or unexplained factors, had closed fallopian tubes, or were smokers. This study did not include patients using frozen oocytes, surgically retrieved sperm, or undergoing an in vitro fertilization cycle (IVF). Finally, control subjects (n = 33) were selected from female candidates undergoing ICSI with male factor infertility based on sperm parameters. Following the World Health Organization guidelines, semen samples were analyzed^58^. The case subjects (n = 54) were selected from subfertile women undergoing ICSI with female factor infertility, including four subgroups; endometriosis (13 patients), PCOS (16 patients), ovarian dysfunction (17 patients), and uterine and cervical lesions (8 patients).

### Blood biochemical assay

The blood serum samples from each patient were collected on the second day of the menstrual cycle and stored at − 20 °C until the serum hormone levels were measured by ELISA procedure. Several hormones, including: Follicle Stimulating Hormone (FSH), Luteinizing Hormone (LH), Estradiol (E2), Prolactin (PRL), Thyroid-stimulating Hormone (TSH), and Anti-Mullerian Hormone (AMH), were conducted using enzymatic kits (Pishtazteb Kit, Iran) according to the manufacturer's instructions. The intra-assay Within-run precision was determined by replicating the determination of three different samples of known concentration in one assay. The coefficient of variation (CV%) was calculated by dividing the standard deviation by the mean into percentage terms. The intra -assay coefficient of variation (CV) was respectively: 7.16% for AMH (mean: 3.35 ng/ml), 2.9% for FSH (mean: 7 IU/L), 4.9% for LH (mean:6.1 IU/L), 3.8% for PRL (mean:13.82 ng/ml), 3.9% for TSH (mean: 4.35 mIU/l), and 5.3% for E2(mean: 37.4 pg/ml).

### The ovarian stimulation protocols

In the control and case groups, controlled ovarian hyperstimulation was performed using gonadotropin-releasing hormone (GnRH) stimulated with GnRH antagonists. Ovarian stimulation was initiated by daily administration of recombinant FSH (Gonal-F; Merk Serono, Germany), starting on day 3 of the menstrual cycle and continued until two follicles with a diameter of 14–15 mm were observed. The administration of GnRH antagonist (Cetrotide; Merk Serono, Germany) was started and continued until at least two follicles with a diameter of 18 mm were observed. Finally, the maturation of oocytes was triggered by injecting human chorionic gonadotropin (hCG, Pregnyl; IBSA, the Netherlands). Approximately 34–35 h after hCG injection, the cumulus-oocyte complexes (COCs) were picked up under ultrasound guidance and short-term anesthesia. There were relatively few variations in the total gonadotropin dose and the number of days of treatment for the ovarian stimulation protocol.

### Collection and preparation of serum, FF, and CCs samples

2 ml of blood from each patient was taken before follicle aspiration on the day of ovum pickup. Then it was centrifuged at 3000 g at 5 °C for 10 min to collect the serum. The serum samples were kept at − 80 °C until miR-21 expression was measured using Real-Time PCR.

Follicular fluid (FF) was collected separately from each follicle during ultrasound-guided transvaginal retrieval. In addition, blood-contaminated or unclear FF was not included in the study. Separately collected FF was placed in sterile tubes and centrifuged for 10 min at 3000 g at 5 °C. The supernatant was kept at − 80 °C for the measurement of miR-21 using Real-Time PCR.

The COCs retrieval was performed utilizing transvaginal ultrasound and a lumen needle (Wallace, ONS1733, CooperSurgical, Inc.). They were collected immediately from FF aspiration. The CCs for each oocyte were removed by manually aspirating into a Pasteur pipette and enzymatically using Hyaluronidase (ART-4007, SAGE In-Vitro Fertilization, Inc.). Each individual's CCs were kept in a tube at − 80 °C to measure miR-21 expression.

### Assay of oocytes and embryos

The oocytes were evaluated for signs of meiotic maturation after they were separated from the CCs. They were examined for the cytoplasmic granulations and presence of the first polar body using a stereomicroscope (Nikon, SMZ 1000) and an inverted microscope (Olympus, IX53). The oocytes were divided into germinal vesicles (GV), metaphase I (MI), and metaphase II (MII). The GV oocytes were defined as oocytes with a visible nucleus. Oocytes determined as MI have no polar body, and those described as MII have a spherical shape and a polar body. A mature oocyte was defined as one in metaphase II and injected with a single sperm (ICSI). Following that, each one was put into a droplet of Sage 1-Step medium (67020010A, SAGE In-Vitro Fertilization, Inc.), covered with mineral oil (ART4008-5P, SAGE In-Vitro Fertilization, Inc.), and kept in an incubator at 37 °C under 5% CO2 and N2 89%, respectively (Esco MIRI® Multiroom incubator).

Fertilization results were assessed by the appearance of two pronuclei (2PN) and two polar bodies at 16–18 h after the ICSI procedure. Embryo quality was evaluated on alternate days. On day 3, each embryo was given a grade based on its cell count, the regularity of its blastomeres, the degree of cytoplasm fragmentation, and the thickness of its zona pellucida^59^. The term "A-grade embryos" was used to describe embryos that had equal blastomeres and no cytoplasmic fragmentation. Each person's data included the number of oocytes obtained, the number of GV, MI, and MII oocytes, oocyte maturity (the ratio of the number of metaphase II oocytes to the total oocytes in percentage), the number of obtained embryos, the number of A-grade embryos, and the good quality embryos (the ratio of the A-grade embryos count to the total embryos in percentage) was noted in both groups, and statistical analysis was performed on it.

### RNA isolation, cDNA synthesis, and real-time PCR assays

Purifying miR-21 from all samples was performed using a commercial kit (Karmania Pars Gene, Kerman, Iran). Briefly, the samples were incubated with lysis buffers A, B, and C for 5 min. Next, the tubes were centrifuged at 12,000 g for 5 min, and the supernatant was collected and moved to the new RNase-free tubes. The precipitation buffer was added, and after incubation for 10 min at − 20 °C, the tubes were centrifuged at 12,000 g for 5 min. The supernatant was disposed of, and the washing buffer was added and centrifuged at 8000 g for 8 min. Finally, the supernatant was disposed of, and after incubation for 10 min at room temperature, the RNA was extracted with 20 µl of DNase/RNase-free water. The extracted miR-21 was converted to cDNA using a particular kit (KPG-cDNA kit, Kerman, Iran) immediately, according to the manufactured kit. The synthesized cDNA was stored at − 80 °C to perform further investigation.

Real-time polymerase chain reaction (RT-PCR) was carried out to determine the levels of miR-21 using a commercial kit (KPG-RTmiR-21, Karmania Pars Gene Company, Iran) and Real-Time PCR equipment (Rotorgene 6000, Qiagen-Corbett Research) following the manufacturer’s instructions. The sequences of miR-21 were as follows; forward primer: 5′-GTATACTAGCTTATCAGACTG-3′ and reverse primer: 5′-GTGCAGGGTCCGAGGT-3′ (NCBI Accession No: NR_029738.1). The relative quantification was done by measuring the increased fluorescence light due to SYBR Green bonding. The Real-Time PCR reactions were run in 20 μl, including 10 μl of SYBR Green Master Mix, 1 μl of each primer (forward and universal reverse primer in 10 μM concentration), 5 μl of cDNA, and 3 μl of deionized water. Cycling parameters were established: 95 °C for 4 min to activate the enzyme, 40 cycles of 95 °C for 13 s, and 60 °C for 30 s. The miRNA expression was normalized by RNU6 with the following sequences; F: 5′-CTCGCTGGCAGCACA-3′ and R: 5′-AACGCTTCACGAATTTGCGT-3′. The relative expression of miRNAs was determined using the ΔΔCT method. The raw data was analyzed using the 2^−ΔΔCt^ formula^60^.

### Statistical analysis

SPSS version 23 was used for the statistical analysis. The variables were compared by an independent t-test between the case and control groups. Analyzing the data among two groups of samples (serum, FF, and CCs) and case subgroups was done using one-way ANOVA followed by the Tukey HSD post Hoc test. The correlations between the data in each group were examined using Pearson's correlation test. The outcomes were displayed as the mean ± SEM. A *p* value of less than 0.05 was considered statistically significant.

### Ethical approval and consent to participate

The Ethics Committee of the Islamic Azad University- Parand Branch approved and oversaw this study (IR.IAU.VARAMIN.REC.1399.035). All participants or their legal guardians gave written informed consent for both study participation and publication of information. No individually identifiable health information/image is included in the manuscript.

## Data Availability

The datasets used or analyzed during the current study available from the corresponding author on reasonable request.
